# Epistemic Trust in Scientific Experts: A Moral Dimension

**DOI:** 10.1007/s11948-024-00489-x

**Published:** 2024-05-24

**Authors:** George Kwasi Barimah

**Affiliations:** Leibniz University Hannover, Institute of Philosophy, Lange Laube 6, 30159 Hannover, Germany

**Keywords:** Epistemic trust in science, Epistemic responsibility, Moral obligation, Experts, Non-experts, Science communication

## Abstract

In this paper, I develop and defend a moralized conception of epistemic trust in science against a particular kind of non-moral account defended by John (2015, 2018). I suggest that non-epistemic value considerations, non-epistemic norms of communication and affective trust properly characterize the relationship of epistemic trust between scientific experts and non-experts. I argue that it is through a moralized account of epistemic trust in science that we can make sense of the deep-seated moral undertones that are often at play when non-experts (dis)trust science.

## Introduction

Trust has a moral dimension. When we invest trust in others, we make ourselves vulnerable to their betrayal. Moreover, those who invite our trust make commitments to do certain things for which we trust them for. When trustees fail to do what they commit to do, we often adopt a moral attitude of blame towards them. In the literature on epistemic trust in science, it has been argued that the relevant notion at play when scientists depend on their colleagues for relevant knowledge is trust and not mere reliance, mainly because of the values involved in inductive risk considerations made by scientists (Wilholt, 2013). In the context of science communication between experts and non-experts, some philosophers of science tend to think that we should avoid moralized accounts of trust talk in science, because the everyday sense of trust, analyzed in assurance views of testimony, has misleading ethical demands. I disagree with those who make such claims. I shall argue in this paper that a non-moral account of trust in science fails to make sense of the deep-seated moral undertones that characterize non-experts (dis)trust in experts.

Additionally, I shall argue that non-epistemic value considerations, non-epistemic norms of assertion and the assurance view of testimony properly capture laypersons’ trust in experts since these provide laypersons with second-order ways of assessing scientific testimony. As Anderson (2011) argues, non-experts decide what to believe by deciding whom to believe. In this case, non-experts resort to an assessment of the person providing the scientific testimony and not science as an institution, per se. Whilst a non-moral account of trust in science focuses on the norms governing the institution of science as the proper focus for non-experts’ trust in scientific testimony, a moralized account locates the proper focus of trust to be the moral character of scientific experts providing scientific testimony. One of the main proponents of an institutional version of a non-moral trust in science account is John (2015, 2018), who alludes to Nickel’s (2013) view that,

our entitlement to defer to scientific expert testimony is ‘norm-based’, not ‘assurance-based’; we learn from scientists via an assumption that, in virtue of certain sorts of social institutions, scientists’ claims are governed by an epistemic norm, rather than via reliance on their ‘assurance’ for the truth of what they say. (John, 2018, p. 77)

In this paper, I shall defend the moralized trust account of epistemic trust in science against criticisms from John (2018). In order to do this, I shall divide the paper into four sections. The first section shall spell out the nature of epistemic trust in science. The second section shall develop an account of the moral dimension of epistemic trust in science by building on the work in philosophy of trust. In the third section, I apply the philosophical analysis in the second section to three cases of interaction between experts and non-experts, namely; a doctor and patient relationship, the relationship between a researcher and a potential research participant and a public health expert and public relationship. I then argue, in the fourth section, against John’s (2018) view that the putative norms of sincerity, honesty and transparency are not essential for ethical science communication.

## Epistemic Trust in Scientific Experts

In his seminal paper on the role of trust in acquiring knowledge, Hardwig (1991) makes the point that there are several problems with institutional safeguards, such as peer review and replication, as the sole reliable means of detecting and deterring fraudulent scientific research. In addition to institutional safeguards, Hardwig argues for a refocusing of our attention on the ethical conduct of individual scientists who engage in research, with his call for the inclusion of research ethics courses within scientific training. This shift away from a sole dependence on institutional measures to a further consideration of the character of individual scientists is continued by Wilholt (2013), who makes the point that conventional standards in science are limited because we cannot standardize every methodological decision within the research process, since such decisions are made deep within the research process. He, therefore, argues for a moralized conception of epistemic trust in which scientists trust the moral character of their colleagues to act from the right value judgements. By epistemic trust in science, Wilholt (2013, p. 248) means the following:

…there arises the possibility of an enhanced kind of epistemic reliance—reliance based on the presumption of shared ideas about the values of true results and the dangers inherent in errors. This kind of reliance presupposes much more than just that other scientists work dependably and professionally, in keeping with the rules of the trade. It presupposes that they have the right attitude towards what they are doing—an attitude whose absence might be considered not just regrettable but to a certain degree blameworthy.

Wilholt’s explication of epistemic trust in science brings to the fore value considerations involved in investing well-placed trust in one’s colleagues. Thus, an assessment of the trustworthiness of a colleague within the scientific community should involve an assessment of competence as well as an assessment of shared value commitments regarding the seriousness of error.

Discussions of epistemic trust in science have been well articulated in the context of scientific collaboration and intra-scientific testimony (Hardwig, 1985, 1991; Gerken, 2015; Rolin, 2015; Wagenknecht, 2015; Kukla, 2012). The underlying idea behind scientific collaboration and the sharing of knowledge within science is that the increasing specialization within the sciences, arising from time and resource constraints, make it practically impossible for a single scientist to acquire all the relevant knowledge required to undertake a research project. Hence, there is the need to depend on the expertise of other scientists. Trusting one’s colleagues for important information, which one lacks, is the most prudent thing to do if one wants to achieve epistemic success (Rolin, 2015; Hardwig, 1991). However, the act of trusting one’s colleagues for important information involves an assessment of their trustworthiness. This is because without well-placed trust, one has less confidence in the knowledge received from one’s colleagues.

Apart from value considerations which Wilholt (2013) considers as vital for epistemic trust in science, what other considerations should one think of when assessing the trustworthiness of collaborators? Answering this question, Fricker (2002) asserts that epistemic trust in other scientists requires one to have an empirical warrant of their trustworthiness. For Fricker, this empirical warrant can be established within the framework of the institution of science when she posits that,

scientists’ basis for trusting each other lies in their knowledge of each other’s commitment to, and embedding within, the norms and institutions of their profession. Unreliability is likely to be subsequently discovered and highly penalised in such a setting, and this gives one strong empirical reason, amongst others, to expect informants to be trustworthy. (Fricker, 2002, p. 383)

In the same light, Margitay (2021) suggests that the norms of competence, conscientiousness and honesty are intrinsic epistemic values in science and should therefore provide a justification for scientists to accept the claims of their colleagues. Rolin (2015), on the other hand, makes the case that a default assumption of the honesty of research collaborators is an extrinsic epistemic value because it promotes the attainment of truth, without being necessary or an indicator of truth. Contrary to Fricker’s view that scientists must have an empirical warrant for the trustworthiness of other scientists, Rolin (2015) argues that the evidence for character is underdetermined by evidence. As a result of this, trust in the honesty of other scientists is a moral value judgment because it is premised on a moral reason that it is morally wrong to doubt the honesty of group members ‘when one does not have a reason to do so’ (Rolin, 2015, p. 171).

Frost-Arnold (2013) acknowledges that in the prevailing account of epistemic trust within science, it is held that the self-interest of scientists, which constrains them from tainting their reputation due to fraudulent work and the desire to be rewarded for excellent work, motivates their trustworthiness and therefore establishes the rationale for trusting the work of fellow scientists. In addition to the self-interest of scientists which is responsive to inducements and sanctions within the scientific community, Rolin (2017) notes that it is part of the dominant thinking within science that scientists are motivated to be epistemically responsible in their work solely because of advancing knowledge.

Enough about the epistemic trust among scientists, what about the epistemic trust that is invested in science from those outside of science? How are we to conceptualize the nature of the public’s epistemic trust in science? Taking a cue from Wilholt’s (2013) work, Irzik and Kurtulmus (2019) develop what they call the ‘public’s enhanced warranted epistemic trust in science’ which is an enhanced form of their concept of the ‘public’s basic epistemic trust in science.’ According to them, in order for a member of the public (M) to have warranted basic epistemic trust in a scientist (S) concerning a certain scientific claim (P) the following must hold:

(1) S believes that P and honestly (i.e. truthfully, accurately and wholly) communicates it to M either directly or indirectly, (2) M takes the fact that S believes and has communicated that P to be a (strong but defeasible) reason to believe that P, (3) P is the output of reliable scientific research carried out by S, and (4) M relies on S because she has good reasons to believe that P is the output of such research and that S has communicated P honestly. (Irzik & Kurtulmus, 2019, p. 1150)

On the other hand, the public’s enhanced epistemic trust in science requires the following,

(5) When public welfare is at stake, in making methodological decisions regarding the distribution of inductive risks with respect to P, S makes those decisions in agreement with M’s assessments of the inductive risks in question, and (6) M has reason to believe that Condition 5 is satisfied. Basic epistemic trust plus these two conditions yield what we call ‘public’s (warranted) enhanced epistemic trust in science’. (Irzik & Kurtulmus, 2019, p. 1155)

Again, these conditions outline the components of well-placed trust in experts. For Irzik and Kurtulmus (2019), in order to have enhanced epistemic trust in experts, the public must assess whether experts share their assessments regarding inductive risks. They suggest that the public would be in a position to make this assessment by engaging with experts through hybrid forums and in the inclusion of diverse values and groups within a research community. I will refer to much of Irzik and Kurtulmus’ work on the nature of the public’s epistemic trust in science when I examine the various contexts of communication in which experts engage with laypersons in Sect. The Contexts of Science Communication.

## The Moral Dimensions of Epistemic Trust in Experts

Some philosophers of science have already explored the moral dimension of epistemic trust within science, usually involving scientific collaborators and other members within the scientific community (Frost-Arnold, 2013; Rolin, 2015, 2017). For instance, Frost-Arnold (2013) and Rolin (2017) argue that in sharing knowledge with their colleagues, scientists are not only motivated by self-interest or epistemic reasons but are also motivated by moral considerations. For Frost-Arnold and Rolin, it is this moral motivation which gives colleagues the reason to trust each other’s testimony. According to Rolin (2017), scientists are motivated to be epistemically responsible to their colleagues because they do not only see it as an epistemic duty but also as a moral duty. This moral duty for Rolin stems from the respect accorded to one’s colleague as a knower. In what follows, I shall advance an argument for a moral dimension of epistemic trust in science by extending the discussion to the epistemic trust relationship between experts and non-expert members of the public.

I want to make the case that the relationship of epistemic dependence^1^ which characterizes the epistemic trust relationship between experts and non-experts provides a reasonable ground to argue for the moral dimension of epistemic trust in experts. Hardwig (1985), describes epistemic dependence as an appeal to the intellectual authority of an expert. Hardwig (1985) elucidates the nature of epistemic dependence by asserting that “because the layman is the epistemic inferior of the expert (in matters in which the expert is expert), rationality sometimes consists in refusing to think for oneself” (Hardwig, 1985, p. 336). For Hardwig, appealing to those who know more than us is a rational way to acquire true beliefs for our epistemic and practical needs. When it comes to the relationship between novices and experts, Goldman (2001, p. 90) posits that novices do not think of themselves as possessing the relevant knowledge that is needed in a specific domain to adjudicate between rival experts. Moreover, Hardwig (1985, p. 341) states further that, laypersons do not fully understand what constitutes good reasons in the domain of expert opinion. Laypersons also face problems when identifying who the real experts in a particular field are because there are sometimes contradictory expert opinions and problems with ranking of experts. All of these problems that laypersons face when interacting with experts puts them in a very precarious position in which they are vulnerable to a betrayal of their trust, even when they have put some effort into assessing the trustworthiness of experts using various second-order criteria^2^.

The inherent vulnerability of laypersons when they trust certain experts for important information which they require to regulate their life, in the best possible way, is what may be called the vulnerability of epistemic dependence. This tendency of laypersons to have their trust betrayed by experts is often morally charged and this is evidently seen in cases of legitimate distrust of certain experts. Moreover, the vulnerability of laypersons who depend on experts is further exacerbated because of the high status that experts enjoy in society over laypersons. This makes laypersons have less influence on collective decisions that affect them and are thereby left vulnerable to the preferences of experts (Desmond, 2022, p. 588). It is based on this that I claim that experts are morally obligated to be epistemically and morally responsible to non-experts who depend on them for reliable information.

However, this specific kind of moral obligation which is established by an epistemic trust relationship does not apply to all scientists in general, but only to those scientists who act as experts on particular topics by placing themselves in the public context, and who by virtue of using various speech acts, such as telling, advising, reporting or advocating, invite trust from their audience (Goodwin, 2018; Hinchman, 2005a, b). This is usually the case for science policy advisors, public health experts or those scientists who write primarily for the public. At this point, it is important for me to emphasize that all scientists have moral obligations to be responsible for the knowledge they produce (Douglas, 2003). However, when it comes to the nature of epistemic trust one must differentiate between those scientists who have actively sought the trust of non-experts and those who have not. Whilst a scientist who works mainly in a laboratory, and who may not ever have an encounter with the public, has not actively sought the trust of non-experts, the scientist who acts in the capacity of an expert by providing information primarily targeted at a non-expert audience on matters that are relevant for them, actively invites their trust. It is important to note that when it comes to epistemic trust the party that is trusted must be aware of the trust of the trustor and be willing to reciprocate by acting to honour the trust of the trustor.

Thus, the moral dimension of epistemic trust is evidenced by the moral obligation which is ascribed to experts because they have invited the trust of non-experts who have responded to this invitation by trusting them, and a commitment by the expert to reciprocate such trust (Hawley, 2012). According to Nickel (2007), the act of trusting ascribes an obligation to the trustee and such an obligation is expected to motivate performance. Nickel argues that obligation-ascription which is inherent in trust relationships provides a reason for blame when trust is betrayed. I develop this view further by appealing to Faulkner’s (2007) discussion of affective trust. Faulkner provides the following conditions to be in place for affective trust to hold:

A trusts S to Φ (in the affective sense) if and only if: (1) A knowingly depends on S Φ-ing and (2) A expects S’s knowing that A depends on S Φ-ing to motivate S to Φ (where A expects this in the sense that he expects it of S). (Faulkner, 2007, p. 882)

When non-experts trust scientific experts for reliable information, they enter into an affective trust relationship with them in which they expect the expert’s recognition that they depend on her for reliable scientific information to motivate her to provide this information (Almassi, 2012). An analysis of the moral dimension of epistemic trust is incomplete without a corresponding commitment on the part of the trustee to fulfill such trust. However, this commitment is satisfied when a scientist takes on the role of an expert by inviting the trust of non-experts when they direct their claims to them. By engaging in speech acts such as telling, advising and reporting, which is directed at non-experts, the expert is committing to vouch for the truth of what is being asserted and therefore invites non-experts to trust them because of their commitment to the truth (Hawley, 2012, 2019). It is taking on the role of expert and the corresponding commitment to be epistemically responsible for the truth of their claims that establishes a moral obligation to do just that. This commitment to the truth of what is asserted warrants reactive attitudes such as blame and resentment when the expert does not follow through with their commitment (Nickel, 2007).

A similar work in this direction is that of Furman (2020) who discusses the role that emotions play in the public distrust of scientific experts. Furman grounds her argument on the trust relationship by referring to the ‘anticipatory attitude’ of fear and anxiety which precedes some epistemic trust relationships, and the reactive attitudes of frustration and anger which a betrayal of trust evokes in those who have trusted certain experts (Furman, 2020, p. 721). She argues that public epistemic trust in scientific experts is difficult to regain when it is lost because emotions are involved.

In the case of marginalized lay communities, Grasswick (2018) asserts that the betrayal of epistemic trust by trusted scientific experts leads to epistemic trust injustices and harms. According to Grasswick, certain marginalized communities face epistemic trust injustices because of a history of betrayed trust. Grasswick argues that historical abuses of trust prevent members of marginalized lay communities from investing well-placed trust in future trustworthy experts, thereby making members of these communities miss out on the benefits of reliable scientific research. A typical example of epistemic trust injustices and harms among sections of the African-American population can be traced back to a breach of trust during the Tuskegee Syphilis study. Also, skepticism towards new vaccines and clinical trials, especially among communities in Nigeria, can be traced to a historical breach of trust during the Pfizer-Meningitis drug trial case.^3^ These examples of distrust show that people trust scientists to be motivated to perform their obligations towards them because of their dependence on scientists and the scientists’ role commitments. The focus of this section has been to unearth the moral aspects of epistemic trust in experts. The argument here is that by epistemically depending on experts, non-experts are left vulnerable because of the epistemic asymmetry between them and also because of the status asymmetry which makes laypersons vulnerable to the preferences of experts. Given the nature of this vulnerability, I have argued that experts are to be epistemically and morally responsible to non-experts who trust them for reliable information. I have also shown that this responsibility which experts have towards non-experts arises because scientists who act in the capacity of experts have invited the trust of non-experts and by acting as experts for the public have made a commitment to honour the trust invested in them. As Nickel (2007) posits, such an investment of trust in experts is expected to motivate performance, while non-performance often results in negative reactive attitudes. How this moral account of epistemic trust plays out in the interactions between experts and non-experts in both private and public contexts will be the focus of the next section.

## The Contexts of Science Communication

I shall now turn my attention, in this section, to how the moral dimension of epistemic trust plays out in three cases of interaction between experts and non-experts. The cases insub-Sect. Application in the Context of a Doctor-Patient Relationship and Application in the Context of Informed Consent (Researcher-Research Participant Relationship) involve a clearly identifiable expert and a non-expert, while the case in sub-Sect. Application in the Context of Public Health Experts is between an identifiable expert and an unidentifiable audience. John (2015) describes the context of communication in which one’s audience is identifiable as private communication and the context in which one’s audience is unidentifiable as public communication. John (2015) is right in identifying different contexts of communication. However, in both contexts, John (2015) argues for epistemic standards as the sole constitutive norm of science communication which experts should be concerned about.^4^ Given the moral analysis of the epistemic trust relationship in Sect. The Moral Dimensions of Epistemic Trust in Experts, I do not think this is plausible. I challenge John’s view in more detail in Sect. Implications for Norms of Science Communication by highlighting non-epistemic norms of science communication as equally relevant, especially in non-experts’ epistemic trust in experts.

### Application in the Context of a Doctor-Patient Relationship

In the doctor-patient context, the doctor acts as an expert providing information about a patient’s health. The patient in this case is the non-expert depending on the doctor for relevant health information. Patients tend to trust their doctors for several things, some of which include confidentiality, prescribing a cure for their disease or performing a surgery well. However, not all such trusting is epistemic trust. A typical case of epistemic trust in the doctor-patient relationship is trusting a doctor for a diagnosis. In this case, the patient epistemically depends on the doctor for accurate and reliable information about the state of her health, and the doctor presents herself as having the expertise to provide this information. By presenting herself as an expert and employing speech acts of telling and advising, the doctor invites the trust of her patient (Hinchman, 2005a, b) and commits to do what she is trusted for (Hawley, 2012, 2019).

Similarly, Goodwin (2018, p. 18) notes that “The responsibility for the truth that the communicator has undertaken in saying something to the audience makes her vulnerable (to criticisms), and her open acceptance of that vulnerability gives her audience a good reason to trust what she is saying.” In this case, an epistemically dependent relationship is forged between the doctor and patient such that the patient trusts the doctor as an expert and in so doing ascribes moral obligations to her to provide accurate information. In such a trust relationship, it is not uncommon to praise the doctor for a right diagnosis and to blame or even resent her for a wrong one (Nickel, 2007). Moreover, as Goodwin (2018) notes, acting as an expert authority or an advisor comes with certain responsibilities. For instance, in the case of a doctor exercising expert authority in a clinical context, the doctor undertakes the responsibility to ‘speak as an expert - and only as an expert.’ In most cases, doctors also perform the role of advisors and in doing so they undertake the responsibility to help their patients with their concerns. (Goodwin, 2018, p. 19). Goodwin (2018) makes the case that when employing the speech acts of exercising authority and advising, the audience (in this case patients) may have legitimate reservations about the intentions of the doctor. As a result of this, Goodwin (2018) argues that experts must consider what they are doing when communicating with their audience, they must be sensitive to the legitimate reservations or distrust that comes along with the kind of speech act they are engaged in and use communicative features to demonstrate to the audience that they can be trusted. The patient takes the fact that the doctor takes on the responsibility (and associated vulnerability to criticisms) of exercising authority and advising to be a good reason to trust the doctor.

Faulkner’s (2007) analysis of affective trust will work in this case of epistemic trust as well. To recap Faulkner’s notion of affective trust again, “A trusts S to Φ (in the affective sense) if and only if: (1) A knowingly depends on S Φ-ing and (2) A expects S’s knowing that A depends on S Φ-ing to motivate S to Φ (where A expects this in the sense that he expects it of S)” (Faulkner, 2007, p. 882). Faulkner’s notion of affective trust is at play in the doctor-patient case because in most cases conditions (1) and (2) hold for patients trusting a doctor for an accurate diagnosis. They knowingly depend on the doctor to provide this information and they expect the doctor’s recognition that they depend on her to motivate her to do so. A lack of motivation on the part of the doctor to provide relevant health information to the patient, after the patient has openly shown dependence (by seeking the doctor’s help), is bound to necessitate feelings of resentment if the doctor does not provide justification for her lack of motivation.

Moreover, epistemic trust is warranted and enhanced in this context if the values of the patient concerning the seriousness of an erroneous diagnosis and the benefits of a true one aligns with that of the doctor’s (Irzik & Kurtulmus, 2019; Wilholt, 2013). Bennett (2022) provides a slightly different account of enhanced epistemic trust in experts, which he calls recommendation trust. According to Bennett’s (2022) view, in order for recommendation trust to be in place, the patient must have good reasons to believe that the doctor has good reasons to believe that her recommendation aligns with the patient’s values.

### Application in the Context of Informed Consent (Researcher-Research Participant Relationship)

The relationship between researchers and potential research participants is not usually discussed as an epistemic trust relationship, but I suggest that it is. The process of obtaining informed consent in health-related research is traditionally seen as a way of respecting the autonomy of research participants. However, I suggest that apart from the ethical component of respect for persons there is the added component of epistemic trust. One way in which this relationship is one of epistemic trust is that potential research participants depend on researchers for the truth and reliability of the information concerning the intended research, written in the informed consent document. In most cases, potential research participants lack expertise about the intended research and do not consent to the research because they understand how the research is going to work, but because they trust the researcher who has presented herself as an expert (Manson & O’Neill, 2007).

Some of the speech acts performed when presenting information about the research to potential research participants include, telling, assuring, requesting, and advising (about the risks and benefits of the research). These speech acts performed by researchers are intended to invite the trust of potential research participants. Again, by engaging in these speech acts, potential research participants take it that the researchers have factored in their vulnerability to criticisms, if things should go wrong, and this provides potential research participants a good reason to trust (Goodwin, 2018). More specifically, when it comes to the speech act of advising potential research participants about the potential risks and benefits of the research, Goodwin (2018, p. 19) suggests that researchers should avoid telling and focus on the concerns of the potential research participant while suppressing their own. The goal of advising in the context of research is to assist potential research participants to arrive at their own decisions which reflect their interests and concerns (Goodwin, 2018). A consideration of the interests of potential research participants in this way provides them with good reasons to believe that the researchers’ values regarding the seriousness of errors and benefits of true results aligns with their own (Irzik & Kurtulmus, 2019; Wilholt, 2013).

A further concern for the interests of potential research participants, especially in the context of clinical research, requires that researchers do not exploit the therapeutic misconception of potential research participants, to enroll them in studies (de Melo-Martin & Ho, 2008).^5^ Researchers can do this by being honest with potential research participants about the aims of the research and how that is different from the aims of clinical care. In a similar vein, Martin (2008) discusses ‘hope’ language in the description of early-phase research trials and the ways such language might be exploitative. Researchers are not to oversell whatever results the trial might arrive at and whatever benefits the patient might receive by participating in the trial.^6^ Hence, consenting to participate in the research establishes a trust relationship between researchers and research participants. In this trust relationship, researchers take on the responsibility of honoring the trust of research participants by carrying out the research in the manner described in the informed consent document and not to exploit the vulnerability of research participants. According to de Melo-Martin and Ho (2008, p. 203), trusting the goodwill of researchers implies that research participants trust researchers ‘to present all information truthfully and not put subjects at unnecessary risk or exploit them.’ Thinking about informed consent in this way guards against obtaining informed consent merely as a means of ticking one of the boxes of ethical research or meeting the requirements of funding organizations and Institutional Review Boards (IRBs).

### Application in the Context of Public Health Experts

The cases in sub-Sect. Application in the Context of a Doctor-Patient Relationship and Application in the Context of Informed Consent (Researcher-Research Participant Relationship) deal with private communication. In such cases the experts as well as the non-expert is identifiable and it is often straightforward to pinpoint the moral responsibility of the expert who is being trusted for reliable information. What about public communication, where the expert is identifiable, but the audience is not? This sub-section will explore such a scenario involving communication between a public health expert and the non-expert public.

In recent times, there has been a rise of scientists acting in the capacity of experts on various public health issues. The kind of public health experts I have in mind are those who are not appointed by policy makers to provide policy advice, but those who feel the need to contribute their knowledge on particular public health topics to the broader society. Some of these experts are invited by the media to provide expert advice on pertinent issues. Many of such experts have emerged during the COVID-19 pandemic and other public health epidemics in the past. I argue that by presenting themselves publicly as experts, they invite trust from the public by *telling* the public that ‘p’ (‘p’ being a public health information, for example, that wearing a mask protects against COVID-19), *reporting* that ‘p’ or *advising* that ‘p’ (Goodwin, 2018). As Goodwin (2018) avers, the expert who engages in these speech acts undertakes certain responsibilities and the audience takes the expert’s assumption of the role of expert authority, scientific advisor or reporter as a good reason to trust them. Furthermore, the public’s epistemic trust in a public health expert is warranted and enhanced when the public has good reasons to believe that the expert shares their values concerning the negative consequences of making erroneous claims (Irzik & Kurtulmus, 2019; Wilholt, 2013). Much has been discussed in the literature on vaccine hesitancy about the mismatch of values between public health officials and parents when it comes to immunization of children leading to distrust of public health experts.^7^

When a section of the non-expert public trusts the public expert, just by virtue of presenting herself as one, an epistemic trust relationship is forged in which the public ascribes moral obligations to the public expert to be epistemically and morally responsible to the relevant non-expert audience. As witnessed in some countries during the COVID-19 pandemic, public health experts who provided reliable information were praised, while those whose claims proved to be unreliable were resented or even blamed in cases of public misinformation.^8^

The doctor-patient and researcher-research participant scenarios are typically interpersonal relationships and it is easier to identify a trust relationship in these contexts. The public health expert and the public relationship, on the other hand, is a ‘one-to-many relationship’ with the trust relationship being a bit more nuanced. However, I maintain that a moral relationship of trust even holds in this case, although it is weaker than what pertains in a ‘one-to-one relationship’. By virtue of the fact that a scientist presents herself as an expert and goes ahead to make claims which tend to invite trust from a relevant group of non-experts, an epistemic trust relationship is forged when the invitation to trust is accepted. A moral obligation is ascribed to the expert and the public expects the expert to fulfill her role obligations (Nickel, 2007; Hawley, 2012, 2019). This relationship persists irrespective of whether an official contract is signed between them or not. An important reason why this is the case is that the information that a trusted expert provides can bring benefits or harms to her audience, if that claim is believed and acted on.

Moreover, an affective trust relationship as described by Faulkner (2007) is evident in such scenarios. This holds true because, (1) members of the public knowingly depend on the public expert for relevant information and (2) they expect the expert’s recognition that they depend on her to provide this information, to motivate her to do so. Distorting information, misleading or misinforming the public who depend on you for reliable information is bound to arouse feelings of resentment, which is evidence that a moral relationship of trust was already at play. If upon such resentment and blame, the public expert responds by saying she did not ask the public to trust her, the public might respond by saying she should not have presented herself (in the media or other public fora) as an epistemic authority on that topic. However, if the situation turns out differently and the information provided by the public expert is reliable, praise for the expert will be the natural response and she will not refuse such praise on the grounds that she did not invite the trust of the public. It seems intuitive that presenting oneself as an expert and making claims to that effect is an invitation to be trusted and such an invitation has a moral dimension if it is accepted.

Exploring these cases which involve private and public communication is my attempt to elucidate the moral dimension of epistemic trust in practical contexts. All the cases share these features; (1) an expert and non-expert relationship, with non-experts depending on experts for relevant information (2) there is an epistemic asymmetry with non-experts at the vulnerable end of things (3) experts have invited the trust of non-experts through speech acts such as telling and advising (4) non-experts count on experts to fulfill their commitments (role obligations) and to be motivated to honour their trust because they are depending on them and (5) in each of these cases epistemic trust is warranted and enhanced when non-experts have good reasons to believe that experts share their values concerning the negative consequences of error. The analysis in this section shows that we also need to focus on non-epistemic norms of science communication, which I do in the next section.

## Implications for Norms of Science Communication

The moral dimension of the epistemic trust relationship between experts and non-experts does not only entail the moral obligation of experts to be epistemically responsible to non-experts by providing them with reliable information, but it also entails the norms involved in communicating scientific information to non-experts. Kelsall (2021) defends trust-based communicative obligations of expert authorities by suggesting that experts who hold positions of public trust are obligated to earn trusting reliance through sincerity, transparency and honesty, in order to legitimize their positions (Kelsall, 2021, p. 302). I, on the other hand, focus on the nature of the trust relationship, the epistemic dependence of non-experts on experts and the resulting moral obligations which this relationship of dependence fosters, especially when experts have invited trust because they are acting in the capacity of epistemic authorities.

There are certain norms which govern the way we transmit information to others. In philosophy of language the norms which have been suggested to govern assertions are the knowledge, truth and justification (factive) norms (Williamson, 2000; Turri, 2013) and belief or non-factive norms (Lackey, 2007). These norms govern the person providing the information and are generally characterized as epistemic norms. However, there are ethical or other-regarding norms which also govern what we say to others. Other-regarding norms such as honesty (Keohane et al., 2014), sincerity (Williams, 2002) and care (Grasswick, 2018) have also been articulated. These norms are characterized as other-regarding because they do not contribute directly to the process of inquiry. What they do is that they acknowledge the dependence of the recipient of information on the speaker and the implications of what is being communicated on the recipient. Thus, the speaker adopts an honest or sincere stance towards the audience for their epistemic as well as their general wellbeing. Rolin (2015) for instance, considers honesty to be a moral value which is neither necessary nor indicative of truth, but promotes the attainment of truth and thus serves as an extrinsic epistemic value.

The epistemic norms of truth, belief and justification relate more to the content of what is said rather than who it is said to. This point matters because, if there were no audience, we would still expect a claim to be true and well justified by the body of evidence. As Grasswick (2018, p. 76) puts it,

While the competency requirement focuses on the testifier’s relationship to the knowledge in question (epistemic), the sincerity requirement emphasizes the ethical dimension of successful testimony practices; it signals a relationship between the speaker and the hearer, and an attitude toward the knowledge recipient.

I hold that experts should be morally responsible to non-experts who trust them for knowledge because of the moral obligation which the epistemic trust relationship establishes. Experts are not only morally obligated to be epistemically responsible to non-experts, but must also be morally responsible to them by being sincere, honest and transparent in their communication.

My conception of the epistemic trust relationship between experts and non-experts runs contrary to the view of John (2018) who does not envisage a moral aspect of epistemic trust. According to John, non-experts learn from experts through the following process:

The sociological premise: Institutional structures are such that the best explanation for the factual content of some claim (made by a scientist, or group, or subject to some consensus) is that this claim meets scientific ‘epistemic standards’ for proper acceptance.

And, second, the epistemological premise: If some claim meets scientific epistemic standards for proper acceptance, then I should accept that claim as well (John, 2018, p. 77).

John’s characterization of the way non-experts learn from experts leads him to suggest that the only ethical requirement which should guide expert communication with non-experts is to avoid wishful speaking. Wishful speaking according to John (2018, p. 84) is “communicating ill-established claims for non-epistemic ends.” What this means in essence is that, the only ethical concern for experts should be epistemic (i.e., making claims that are well-established based on the evidence). John goes further to say that a concern for norms such as sincerity, honesty, transparency and openness if adhered to by experts, in some cases, can be detrimental to non-experts. For John, the main task of experts is to promote the epistemic interest of their audience and in so doing the norms of sincerity, honesty, transparency and openness can be sacrificed, if they stand in the way of this goal. I acknowledge that there are particular situations where complete honesty and transparency in research may have obvious negative consequences that are not in the public’s interest. For example, scientists are not to be transparent about dangerous knowledge, which may include knowledge of how to produce an atomic bomb or in dual-use research, all of which pose a great threat to the lives of people if this information gets into the wrong hands. Also, scientists are not to be transparent about the identity of research participants involved in their study. In such cases, there are legitimate reasons to keep this information or knowledge secret and the public trusts scientists to keep such knowledge secure. However, we cannot generalize from this to discard the putative communicative norms in their entirety.

John (2018) argues against these putative norms of communication by focusing on anthropogenic climate change as a case study. The case of climate change is particularly interesting because of various non-epistemic interests at play, especially for those who would want to deny that anthropogenic climate change is taking place because of the implications this will have on their economic interests. John (2018) identifies two main factors that will make a sincere, honest, transparent and open communication of scientists on interest-driven public issues such as climate change dangerous.

First, John suggests that the public has a false folk philosophy of science, which he describes as “extremely idealised and unrealistic normative models of scientific inquiry” (2018, p. 81). For example, the public might believe that science delivers certainty when in fact uncertainties are commonplace in science, or that scientific results are infallible such that revising certain previously accepted scientific results is seen as bad science, when in fact revision of certain scientific claims based on new evidence is the norm in science.

Following from this, the second threat which John (2018) identifies are ‘merchants of doubt’^9^ who may take advantage of a false folk philosophy of science to distort justified scientific claims that have been communicated honestly and transparently. John cites the Climategate scandal to illustrate that transparency about uncertainties in climate science research can be manipulated by climate change denialists to serve their non-epistemic interests. I find this example that John cites as an argument against transparency to be problematic, primarily because it was not the case that climate scientists were transparent in the first instance. The scientists were not open about their work (not that they had anything to hide or were being manipulative), but it was climate change denialists who leaked emails of the scientists and who went on to sow doubt in the public domain about the work of the scientists. However, if the climate scientists, working in the University of East Anglia, had made their work transparent to the public from the get go and explained how they weighed evidence to justify their conclusions, there would be no need for damage control after climate denialists had come in to expose and distort their work. What the Climategate case teaches us is that greater transparency, not less, is essential for trustworthy science communication.

I am of the view that John does not draw this conclusion because a moral analysis of the epistemic trust relationship between experts and non-experts is ignored in his view of the way non-experts learn from experts. John’s analysis of the way non-experts learn from experts is oversimplified and it flies against much in the literature which investigates the nature of non-expert’s epistemic trust in experts. However, it is based on John’s two premises of how non-experts learn from experts that he argues against the putative communicative norms.

John’s (2018) view of how non-experts learn from experts is oversimplified in the following ways. First, in deciding which expert to trust, especially in controversial scientific matters such as anthropogenic climate change, safety of vaccines and Genetically Modified Organisms (GMOs), non-experts do not only look out for claims that meet scientific ‘epistemic standards’ for proper acceptance, but they also consider whether the scientist or group of scientists making a claim have their interest at heart. Non-experts, before deciding whom to trust, would want to know whether the expert(s) is honest (Anderson, 2011), have a good will towards them (Baier, 1986), are motivated to be ethically and epistemically responsible when they recognize that non-experts are depending on them for knowledge (Almassi, 2012), or whether experts share their values concerning judgments of inductive risks (Irzik & Kurtulmus, 2019), as I have argued for in the previous sections.

Moreover, a more complex understanding of how non-experts learn from experts, especially about scientific matters which non-experts care about, shows that non-experts are not only concerned with epistemic considerations, but ethical and value considerations as well. My analysis of the affective trust relationship between experts and non-experts shows that there are some expectations that non-experts have of experts and this should make experts concerned not only about communicating well-established claims, but to do so in a sincere, honest, and transparent way.

An overly exaggerated concern for the epistemic interest of non-experts without maintaining a moral concern for them leads to an extreme form of epistemic paternalism^10^. This aggravated form of epistemic paternalism is often characterized by a disrespect of the autonomy of non-experts to make informed decisions after a transparent and honest presentation of the facts have been provided to them. A consequence of John’s argument is his claim that “communicating well-established claims may sometimes require our actual assertions are dishonest” (John, 2018, p. 85). John makes this point especially in the context of climate change policy where communicating all other uncertainties might lead to delay or no political action from policy makers. John suggests, in this case, that while it is dishonest to communicate only the well-established claim without the uncertainties, the experts will be ‘well-leading’ instead of misleading policy makers. Such a forceful need for experts to get politicians to take particular decisions on issues which affects the public blurs the line between ‘academic advice and political decision-making’ (Hodges et al., 2022, p. 252; Van Dooren & Noordegraaf, 2020).

Furthermore, as Carrier (2022, p. 5) suggests, doubt in the public arena about scientific policy advice arises because some sections of the population are “suspicious about the value judgments passed by experts and are afraid of a technocratic rule.” This is the more reason experts should be transparent and honest about their value judgments and about uncertainties to avoid taking too much decision-making power away from publicly elected policy makers. Returning to the arguments I have made about the role obligation of experts when they engage in different speech acts directed at the public, Goodwin (2018, p. 19) notes that scientists who engage in the speech act of exercising expert authority are to accompany their statements with needed caveats, limitations, hedging and to communicate uncertainties, since that will promote trust overall. She makes this claim based on empirical work which has shown mixed results when it comes to the communication of uncertainties leading to trust or distrust. However, studies by Fischhoff (2012) and Fischoff and Davis (2014) show that “overall, empirical studies provide confidence that lay audiences are able to understand well-communicated scientific uncertainties.” (Goodwin, 2018, p. 22, footnote 5).

This notwithstanding, what should be our response to John’s (2018) legitimate worry of communicating scientific uncertainties within an environment of a false folk philosophy of science, with the potential for distortion by agnotological threats? I believe the way to go is to communicate scientific uncertainties and probabilities honestly and transparently to policy makers, while emphasizing the evidential superiority of some particular evidence over others.^11^ This approach will help to fulfill the scientific advisors’ dual role of promoting objectivity and providing guidance for policy makers (McKaughan & Elliott, 2018). Similarly, Carrier (2022), in his proposal for what good scientific policy advice to politics should look like, argues for a version of the value-free ideal in policy advice in which experts “elaborate a plurality of policy packages that envisage the implementation of different social goals.” In this case, scientific policy advisors will not be seen to be illegitimately imposing certain values on the public and this may make the advice given more trustworthy (Carrier, 2022, p. 5). On a more general level, I am of the view that it will take a concerted effort of different agents to correct a false folk philosophy of science and to deal with the agnotological threats to reliable information acquisition by the public. I suggest sharing epistemic and moral burdens among scientific experts, science journalists and philosophers of science. When these various agents take their duty to the public to be an epistemic as well as a moral one, we can yield some dividends from this collective endeavour.^12^ The suggestions I make below obviously need further critical discussion and elaboration which cannot be adequately done here, but I think these first steps at developing a solution are worthy of our attention.

i. Experts should not just tell the public how justified their claims are but should engage opposing views which they consider ill-established.
ii. After this is carefully done, the decision to be taken (concerning what to do) must be left to the public and their policy makers. Experts would have fulfilled their epistemic and ethical roles if they just do this.
iii. Science journalists should help the public unravel value influences of scientists (Elliott, 2019); they should make a conscientious effort to balance balanced reporting and reliable reporting (Gerken, 2020) and make use of their investigative training to uncover unfounded scientific claims (Christensen, 2008).
iv. Philosophers of science must dedicate some time to engaging with the public if we are to correct a false folk philosophy of science. Philosophers of science understand the inner workings of science better than most people. They can explain the methods of science, the value-ladenness of science and the fallibility of scientific findings which are justified to a higher or lower extent. In effect, there must be a lot of effort invested in proper science literacy (Kovaka, 2021; Douglas, 2021).

This section has sought to criticize John’s (2018) view that epistemic considerations are what is needed when it comes to communicating controversial topics such as climate change and as such, we should do away with norms such as sincerity, transparency, honesty and openness. My response to John has been that these non-epistemic norms of science communication are equally important and that this stems from the moral force of the epistemic trust relationship. Failure to acknowledge the moral dimension of epistemic trust in experts, in contexts of science communication, rips the notion of trust of its strong moral sense.

## Conclusion

It is important that the moral dimension of the epistemic trust relationship is articulated in science because scientific research has implications for various aspects of society, especially regarding people’s lives and wellbeing. An overemphasis on the epistemic aspects of trust is insufficient to account for the expectations that people have of scientific experts and the moral responsibilities experts have towards non-experts. By using the concept of affective trust as a framework and building on speech act theory in philosophy of language, I have proposed that there is a moral dimension to epistemic trust in scientific experts. According to my account, the moral dimension of the epistemic trust relationship arises when scientists put themselves out in the public domain as experts on certain issues, and correspondingly employ speech acts of telling or advising as a means of inviting the trust of the public. In the event that this invitation to trust is acknowledged and accepted by certain sections of the public, an epistemic trust relationship with moral obligations to be epistemically and morally responsible to the trustors is formed. Failure to honour such trust is morally blameworthy and attracts feelings of resentment, while honouring such trust is praiseworthy and attracts feelings of gratitude.

This paper has been an attempt to spell out a moral dimension of epistemic trust in scientific experts. It has also attempted to show that there are rich benefits to be derived from useful exchanges between philosophy of science and certain aspects of traditional epistemology. Also, I hope I have been able to show that ethics and epistemology are interwoven in an intricate way and this must be further explored in philosophy of science. Finally, this paper contributes to the body of work on the epistemic and moral responsibilities of scientists to members of the society by focusing on the relationship of epistemic dependence and epistemic trust between scientific experts and non-experts.

## Footnotes

1 Hardwig (1985) uses this term to describe the epistemic relationship between non-experts and experts.

2 For detailed discussion of second-order assessment of experts, see Goldman (2001) and Anderson (2011) and for discussions of the limitations of laypersons to fully utilize these second-order criteria in deciding which expert to trust, see Barimah (2022).

3 In 1996 the northern state of Kano, Nigeria, was hit by Africa’s worst Meningitis epidemic. As a result of this, the drug manufacturer, Pfizer, carried out a clinical trial in the affected community. Trovan and Ceftriaxone were the drugs to be tested. Out of 100 children who were given the experimental drugs, 11 of them died. Five of the eleven children that died received Trovan, while the rest of the six received Ceftriaxone. Pfizer was sued and it was later discovered that Pfizer did not have proper consent from parents to use an experimental drug on their children and questions were raised over the documentation of the trial. Pfizer had to pay compensation to the bereaved families (Smith, 2011).

4 John (2015) identifies different obligations of experts when it comes to communicating scientific findings to both a known and an unknown audience. In the former context, the expert is under an obligation to “…consider their audience’s proper epistemic standards for acceptance when setting their own epistemic standards for assertion” (John, 2015, p. 82). John (2015), calls this the floating standards obligation (FSO). In the latter context involving an unidentifiable audience (as the case of the public health expert in sub-Sect. Application in the Context of Public Health Experts will show), John makes a case for experts to observe a norm of fixed, high epistemic standards of assertion primarily because one cannot identify their audience’s proper epistemic standards for acceptance since they are unidentifiable. John (2015) goes on to defend and prescribe an obligation of experts to adopt fixed, high epistemic standards of assertion in both private and public communicative contexts. In his view, if an audience knows that claims made by experts are governed by these standards and not by floating epistemic standards, which can be high or low depending on the audience, ‘they can reasonably assume that they should defer to those claims whatever their practical interests’. (John, 2015, p. 88).

5 Therapeutic misconception is the tendency of research participants to confuse the goals of clinical research and clinical care. Whilst the aim of clinical care is to advance the medical health of a particular patient, clinical research seeks to answer ‘a scientific question in order to provide generalisable knowledge that can help future patients’ (de Melo-Martin & Ho, 2008, p. 202). This conflation by research participants between the goals of clinical care and research often leads to a situation of misplaced trust, in which research participants trust researchers to deliver results for their particular medical condition which is not the aim of clinical research.

6 I thank an anonymous reviewer for articulating this point.

7 See Irzik and Kurtulmus (2019) for a lengthy discussion on the role that value judgments regarding inductive risks played in the MMR vaccine and autism controversy in the UK.

8 In Germany, one of the public experts who rose to public acclaim during the COVID-19 pandemic was Christian Drosten, a virologist, who provided regular podcasts on the implications and actions required to combat the pandemic. (See https://en.wikipedia.org/wiki/Christian_Drosten). On the other hand, Dr. Didier Raoult, a French infectious disease expert who claimed that the anti-malaria drug, Hydroxylchloroquine, could cure COVID-19 was faced with resentment from a section of the public and faced disciplinary action. (See https://www.theguardian.com/world/2020/nov/12/covid-professor-didier-raoult-hydroxychloroquine).

9 See Oreskes and Conway (2010) who coin this term in their book.

10 Epistemic paternalism is the idea that in some circumstances it is justified to interfere with the epistemic pursuit of others in their own epistemic interest, without consulting them. See Goldman (1991); Ahlstrom-Vij (2013); Bullock (2018); and Pritchard (2013) for extended discussions.

11 McKaughan and Elliott (2018, p. 204) suggest a way to do this through a process of ‘backtracking’. They describe how backtracking would work in practice in the following way: “Suppose that a scientist were disseminating information that was debated or subject to multiple interpretations in the scientific community. An initial step would be for her to acknowledge the range of different interpretations available when writing journal articles or speaking with reporters or writing for a popular audience. Another important step would be for her to clarify which interpretations were best supported by the available evidence and which ones were more speculative. Because individual scientists are subject to a range of idiosyncratic preferences and biases, it would be particularly valuable for her to clarify the views of the scientific community as a whole about the evidence for different interpretations rather than focusing solely on her own views.”

12 For all these agents something can be said about the moral dimension of their roles, since a relationship of epistemic trust and dependence characterizes their relationship with members of the public. What I have argued so far about the moral dimension of epistemic trust affects all these agents in the science communication landscape.
